# Supplementary material for: Optimizing environmental enrichment for Sprague Dawley rats: Exemplary insights into the liver proteome
Source: PLoS One. 2024 Apr 18;19(4):e0297497. doi: 10.1371/journal.pone.0297497 (PMC11025844; doi:10.1371/journal.pone.0297497)
Supplement: S1 Table — DHEA: dehydroepiandrosterone, ALT: alanine aminotransferase, AST: aspartate aminotransferase, AP: alkaline phosphatase, LDH: lactate dehydrogenase. (PDF) [file pone.0297497.s001.pdf]

Supporting Information

Variable	Overall<br>N = 24	C<br>Control<br>N = 6	CP<br>Control & Playpen<br>N = 6	LC<br>Large Cage<br>N = 6	LCP<br>Large Cage & Playpen<br>N = 6	Kruskal-Wallis Test<br>p	Bartlett Test<br>p
Baseline weight (g)	728 (700, 755)	726 (683, 748)	732 (709, 766)	720 (681, 750)	734 (710, 750)	>0.9	0.50
Final weight (g)	790 (755, 852)	773 (716, 835)	779 (756, 842)	792 (774, 812)	792 (772, 851)	>0.9	0.39
Weight delta (g)	63 (45, 83)	58 (29, 84)	50 (40, 84)	64 (61, 75)	66 (62, 69)	>0.9	0.48
Urea (mmol/dL)	6.16 (5.91, 6.49)	6.24 (6.04, 6.45)	6.24 (6.16, 6.45)	6.24 (5.99, 6.49)	5.41 (5.04, 5.91)	0.063	0.71
Creatinine (mg/dL)	30.1 (25.6, 35.8)	26.5 (23.0, 37.4)	34.5 (25.6, 36.3)	31.4 (30.3, 37.1)	28.7 (26.1, 31.4)	0.6	0.13
AST (U/L)	111 (97, 126)	110 (105, 118)	112 (105, 113)	114 (98, 143)	108 (91, 126)	>0.9	0.01
ALT (U/L)	104 (92, 157)	100 (92, 110)	181 (166, 198)	106 (96, 122)	88 (70, 101)	0.022	0.009
AP (U/L)	118 (94, 137)	103 (94, 112)	128 (127, 165)	120 (94, 139)	132 (111, 144)	0.3	0.03
LDH (U/L)	182 (106, 254)	85 (74, 94)	182 (173, 266)	354 (198, 486)	188 (149, 194)	0.016	<0.001
Albumin (mg/dL)	35.00 (33.35, 35.58)	35.30 (34.43, 35.50)	33.50 (31.10, 35.40)	36.20 (35.40, 37.30)	34.20 (33.52, 34.72)	0.2	0.45
pH	7.19 (7.16, 7.24)	7.19 (7.15, 7.21)	7.22 (7.18, 7.27)	7.17 (7.11, 7.22)	7.20 (7.17, 7.26)	0.5	0.24
Sodium (mmol/L)	141.50 (141.00, 142.25)	141.00 (141.00, 142.50)	141.50 (140.25, 142.00)	141.50 (140.25, 142.00)	142.50 (141.25, 143.75)	0.6	<0.001
Potassium (mmol/L)	3.75 (3.58, 4.03)	3.70 (3.53, 3.88)	3.70 (3.62, 3.77)	4.15 (3.78, 4.38)	3.80 (3.55, 4.05)	0.6	0.25
Calcium (mmol/L)	1.41 (1.38, 1.43)	1.41 (1.40, 1.43)	1.36 (1.35, 1.36)	1.44 (1.44, 1.45)	1.40 (1.38, 1.41)	0.005	0.71
Chloride (mmol/L)	109.00 (107.75, 110.00)	107.50 (107.00, 108.75)	109.00 (109.00, 109.75)	109.50 (108.25, 110.75)	109.50 (107.50, 111.50)	0.3	0.08
Glucose (mg/dL)	15.87 (14.82, 16.86)	16.76 (16.26, 17.05)	16.82 (15.58, 18.47)	15.57 (15.21, 15.76)	14.76 (13.32, 15.91)	0.2	0.28
Lactate (mg/dL)	16.0 (14.8, 21.0)	18.0 (13.5, 24.8)	16.0 (15.2, 16.8)	21.0 (18.8, 23.2)	14.0 (13.0, 15.8)	0.3	0.015
Corticosterone (pg/mg)							
Baseline	16.98 (14.88, 19.22)	16.98 (15.51, 17.57)	15.32 (13.80, 17.84)	17.84 (15.32, 20.58)	18.32 (15.78, 20.31)	0.5	0.15
3 Months	12.66 (11.31, 14.68)	11.70 (11.17, 12.34)	12.51 (11.50, 16.06)	13.46 (12.63, 13.93)	14.22 (12.74, 15.52)	0.3	0.84
6 Months	16.8 (14.4, 19.0)	13.7 (9.5, 15.1)	16.8 (15.4, 19.8)	16.0 (12.4, 18.7)	18.6 (18.4, 19.6)	0.2	0.25
Testosterone (pg/mg)							
Baseline	1.96 (1.62, 2.10)	1.89 (1.66, 2.02)	1.89 (1.65, 2.02)	2.01 (1.57, 2.28)	2.12 (1.78, 2.25)	0.7	0.57
3 Months	1.64 (1.45, 1.79)	1.54 (1.29, 1.59)	1.58 (1.36, 1.75)	1.80 (1.73, 1.97)	1.63 (1.47, 1.77)	0.5	0.70
6 Months	1.51 (1.35, 1.82)	1.08 (0.99, 1.21)	1.79 (1.55, 1.82)	1.54 (1.40, 1.77)	1.66 (1.55, 1.79)	0.007	0.21
DHEA (pg/mg)							
Baseline	8.38 (6.46, 10.34)	6.85 (5.52, 8.03)	8.31 (6.98, 9.37)	9.19 (7.06, 10.17)	10.37 (9.76, 11.97)	0.2	0.77
3 Months	6.56 (5.43, 7.58)	7.11 (6.57, 8.24)	6.70 (6.37, 7.05)	5.90 (5.31, 7.15)	5.67 (4.97, 7.41)	0.6	0.36
6 Months	3.05 (2.47, 4.05)	2.56 (2.39, 2.61)	4.72 (3.69, 7.21)	3.73 (2.88, 6.02)	2.47 (2.08, 3.13)	0.017	0.01

S1 Table. Baseline and laboratory data of study rats. DHEA: dehydroepiandrosterone, ALT: alanine aminotransferase, AST: aspartate aminotransferase, AP: alkaline phosphatase, LDH: lactate dehydrogenase
